# Graduate Medical Education in Lebanon: Challenges, Support, and Adaptation Amid the Compounding Crises

**DOI:** 10.5334/pme.1721

**Published:** 2025-09-10

**Authors:** Fatima Msheik-EL Khoury, Carine Zeeni, Halah Ibrahim, Frida Atallah, Salah Zeineldine

**Affiliations:** 1 Department of Anesthesiology and Pain Medicine, American University of Beirut Medical Center, Lebanon; 2 International Outreach, Accreditation Council for Graduate Medical Education International, Chicago, IL, US; 3 Graduate Medical Education Office, American University of Beirut Medical Center, Lebanon

## Abstract

**Introduction:**

Graduate Medical Education (GME) systems often face disruptions. In Lebanon, repeated crises over the past several years, including political instability, economic collapse, the COVID-19 pandemic, and military conflict, raise questions about institutional support, clinical residents’ preparedness, and the resilience of the GME system. This study aims to examine clinical residents’ perceived preparedness and competency at graduation, the challenges they faced during compounding national crises, and how training institutions supported them and adapted GME to maintain training and well-being.

**Methods:**

In June 2024, we conducted a cross-sectional survey of graduating clinical residents including fellows and residents, in a large academic medical center in Lebanon. Thirteen questions were developed by a group of program directors from different specialties. Quantitative data assessed residents’ self-perceived preparedness across six ACGME core competencies. In parallel, qualitative data explored the challenges residents faced, institutional interventions, and residents’ recommendations. Descriptive statistics were used to analyze quantitative data. Qualitative analysis of open-ended survey responses was guided by Maslow’s Hierarchy of Needs to understand residents’ challenges, while inductive thematic analysis was used to synthesize the institutional strategies.

**Results:**

127 of 133 (95%) residents and fellows across a wide range of specialties, including medical-surgical specialties, completed the survey. Despite ongoing training disruptions, most residents felt well-prepared in core competencies, including 67% for patient care, 54% for medical knowledge, 57% for systems-based practice, 69% for communication skills, 72% for professionalism, and 61% for Practice-Based Learning and Improvement. Open-ended responses revealed that the crises provided opportunities for residents to develop their skills in the systems-based practice competency domain. Five themes on residents’ challenges emerged: meeting basic needs, ensuring well-being, maintaining family and social life, fostering professional growth and clinical experience, and fulfilling career aspirations. Institutional strategies, particularly through the provision of essential financial support, structured emotional and psychological support programs, faculty and program leadership social support, sustained career support and supervision amid crisis, and career mentorship and support, helped maintain continuity in resident training despite severe challenges. Some challenges were beyond the institution’s resources and control.

**Discussion:**

The unique resilience of Lebanese GME programs despite multiple external disruptions highlights the strengths and vulnerabilities of institutional support systems. While the study identified several significant challenges faced by residents and fellows, it underscored the importance of prioritizing their well-being, fostering a supportive learning environment, and developing crisis-relevant competencies to ensure the continued success of medical education in the face of future challenges.

## Introduction

The integrity of graduate medical education (GME) programs is essential for ensuring that future physicians are well-equipped to provide high-quality care, particularly during periods of uncertainty and crisis. Healthcare systems under strain – whether from natural disasters, infectious disease outbreaks, or sociopolitical instability –frequently encounter significant GME challenges [1]. Historical examples include the Western economic crisis of the 1970s, which reportedly weakened the medical profession by decreasing funding for medical schools [2], and more recent European recessions that stalled residents’ salaries [3]. Decades of war, sanctions, and political instability have severely disrupted Iraq’s once-robust GME system [4]. Similarly, the COVID-19 pandemic caused widespread and enduring disruptions to residency and fellowship training programs across specialties. While numerous studies have explored clinical residents’ experiences and institutional support during the pandemic, data remains limited from settings experiencing prolonged, overlapping crises.

Lebanon provides a unique context to investigate GME resilience amid multiple crises. Since 2019, the country has faced political instability, economic collapse, safety concerns, the COVID-19 pandemic, and military conflict. The economic crisis, marked by hyperinflation and currency devaluation that caused the Lebanese Lira to lose over 90% of its value, has profoundly impacted the healthcare system [5]. These challenges have been compounded by the COVID-19 pandemic [6,7,8], the 2020 Beirut Port explosion, which damaged six hospitals and over 22 healthcare facilities [9,10,11], and recent conflicts at the southern borders that closed over 100 primary healthcare clinics and hospitals [12]. A study on ophthalmology training in Lebanon reported that these crises increased resident stress, anxiety, and depression due to financial strain, disruptions to education, and increased clinical demands [13]. However, there is little research specifically examining the cumulative effects of concurrent crises on Lebanese residents across specialties.

This study explores how Lebanese residents perceive their preparedness and competency at graduation, the challenges they encountered during compounding national crises, and the institutional strategies used to support them. By identifying key adaptations in GME, we aim to inform strategies for strengthening GME resilience in future crises.

## Methods

### Study Design, Setting, and Participants

In June 2024, we surveyed graduating residents and fellows at an academic health center in Lebanon to examine the impact of multiple ongoing crises on their professional training and experiences. The study follows the Strengthening the Reporting of Observational Studies in Epidemiology (STROBE) guidelines [14].

This study was conducted at Lebanon’s largest academic medical center, which sponsors 22 residency and 38 fellowship programs. All residency and two fellowship programs adhere to the ACGME-International (ACGME-I) standards, emphasizing competency-based training across six core domains: patient care, medical knowledge, systems-based practice, interpersonal and communication skills, professionalism, and practice-based learning and improvement [15]. These programs mirror US GME in structure, assessment methods, and policies.

Eligible participants included all final-year residents and fellows, across a wide range of specialties (medical and surgical), collectively referred to as ‘residents’ throughout the manuscript. We targeted this group because the exit survey is routinely administered to graduating residents, allowing us to assess the cumulative effects of training and institutional support throughout their training experience.

### Survey Development and Data Collection

In June 2024, an additional section was incorporated into the existing GME exit survey, which was distributed to all graduating residents. The survey was developed by five program directors across different specialties and piloted for length and clarity by ten residents not part of the graduating class of 2024. Minor stylistic changes were made based on their feedback. The final survey (Supplementary Digital Appendix 1) included two demographic questions on specialty type (e.g. anesthesiology, internal medicine, etc.) and training level (residency or fellowship), and six quantitative items evaluating self-perceived preparedness across ACGME-I competency domains, using a three-point Likert-like scale (1 = unprepared, 2 = prepared, 3 = well-prepared). This scale was adapted from the AAMC Resident Readiness Survey to align with established assessment frameworks [16]. Additionally, five open-ended questions explored: (1) challenges faced during Lebanon’s crises, (2) impacts on education and training, (3) perceived institutional support, (4) recommendations for leadership, and (5) future career plans. The survey was administered online via the LimeSurvey platform, using secure, anonymous tokens issued to each resident to ensure each participant could submit only one response while maintaining confidentiality. Participation was voluntary and anonymous. The survey and all responses were in English, the primary language of medical education in Lebanon.

### Data Analysis

Quantitative data were analyzed using SPSS (version 25). Descriptive statistics, including frequencies and percentages, were calculated to summarize resident perceptions of preparedness across competency domains.

For qualitative data, we employed a theory-informed thematic analysis [17,18], guided by Maslow’s Hierarchy of Needs [19], to synthesize residents’ challenges. We began with a deductive coding process using Maslow’s framework to categorize these challenges into the five domains of physiological needs, safety, love and belonging, esteem, and self-actualization [19]. While these domains provided the structure for initial coding, the theme labels were refined to better align with participants’ language and the contextual nuances of the crises. This approach helped ensure a structured yet contextually grounded presentation of challenges that encompassed both personal and professional dimensions. Inductive thematic analysis was then conducted for responses related to institutional support. These responses were independently coded and synthesized into key categories of institutional strategies.

Coding and thematic analysis:

Two researchers (FM and CZ) independently conducted an initial round of familiarization and coding, identifying key concepts and themes within the qualitative data. They then compared codes, refined them through discussion, and reached consensus to establish a final coding schema. Codes were then grouped into higher-order categories and themes were identified through an iterative process of synthesis and abstraction. To ensure methodological rigor, researchers maintained reflexive journals, documenting their analytic process, positionality and potential biases. Given their dual roles as faculty members and investigators, they critically examined how institutional affiliation might shape interpretations, prioritizing the residents’ own wording and perspectives during theme development.

### Ethical Approval

The study was reviewed and approved by the *anonymized* Institutional Review Board (IRB Reference No. SBS-2024–0276) with a waiver of informed consent, as data were de-identified and retrospective.

## Results

### Quantitative findings

**Figure 1:**
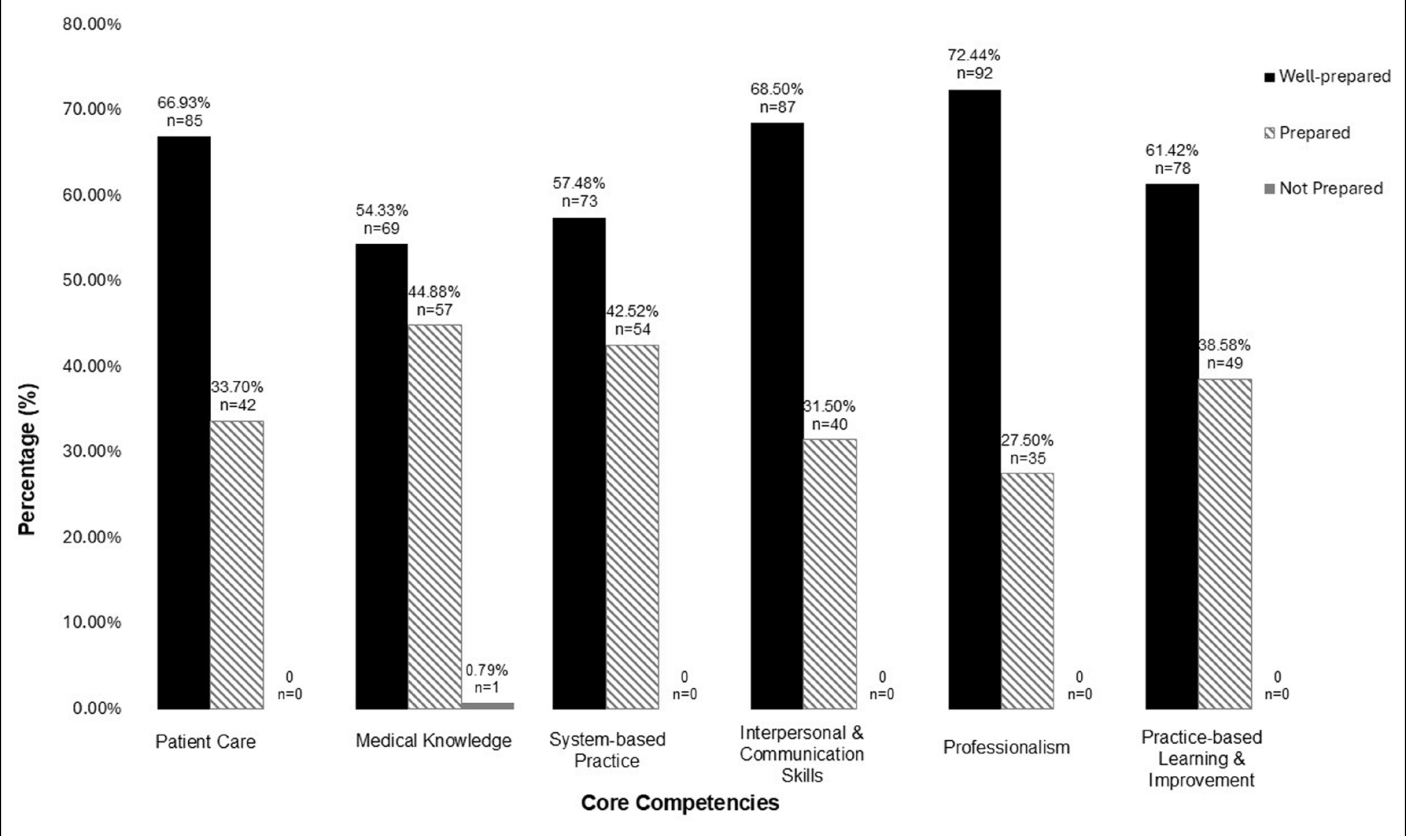
Percentage and Frequency of Preparedness of Resident Survey Respondents across the Six ACGME Core Competencies.

A total of 127 out of 133 residents (95.27%) from medical and surgical specialties completed the survey, with complete data, including all qualitative responses. Participant demographics are detailed in Table 1. Overall, residents reported feeling well-prepared across all six ACGME-I core competencies as detailed in Figure 1.

**Table 1:** Demographic Characteristics of Survey Respondents (N = 127).


VARIABLE	*n* (%N)

**Specialty**	

Anatomic Pathology/Clinical Pathology	3 (2.36%)

Anesthesiology	8 (6.29%)

Dermatology	2 (1.57%)

Diagnostic Radiology	3 (2.36%)

Emergency Medicine	4 (3.15%)

Family Medicine	11 (8.66%)

Internal Medicine	42 (33.07%)

Neurology	5 (3.94%)

Obstetrics & Gynecology	5 (3.94%)

Ophthalmology	2 (1.57%)

Pediatrics	17 (13.39%)

Psychiatry	3 (2.36%)

Surgery – General Surgery	6 (4.72%)

Surgery – Orthopedic Surgery	2 (1.57%)

Surgery – Otorhinolaryngology & Head	2 (1.57%)

Surgery – Plastic & Reconstructive Surgery	2 (1.57%)

Surgery – Urology	1 (0.79%)

Other (orthodontics, radiation oncology, etc.)	9 (7.09%)

**Training Program**	

Residency	99 (77.95%)

Fellowship	28 (22.05%)


### Qualitative findings (summarized in Figure 2)

**Figure 2:**
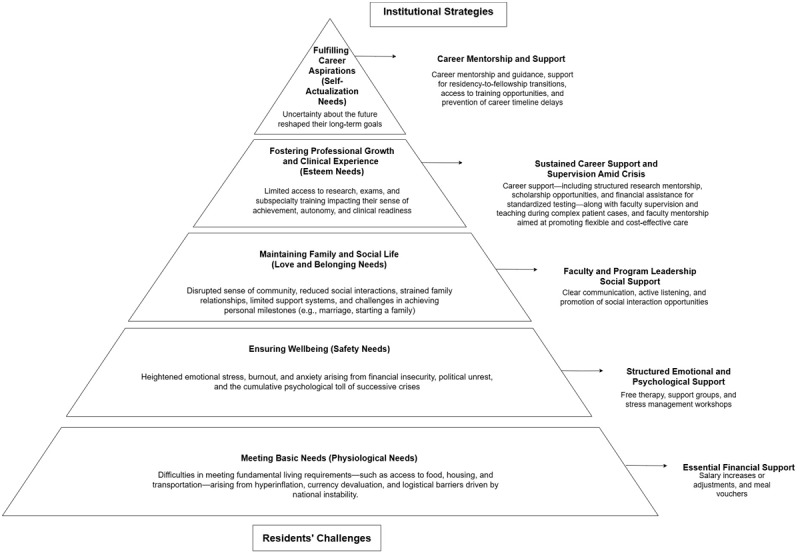
Maslow Domains and the Institutional Strategies.

Residents’ challenges were deductively categorized into five themes according to Maslow’s Hierarchy of Needs: (1) meeting basic needs, (2) ensuring safety, (3) maintaining family and social life, (4) fostering professional growth and clinical experience, and (5) fulfilling career aspirations. Each theme is presented below, supported by illustrative quotes from participants, that reflect on their challenges during the cumulative crises. Institutional strategies were also summarized into 5 themes: (1) essential financial support, (2) structured emotional and psychological support programs, (3) faculty and program leadership social support, (4) sustained career support and supervision amid crisis, and (5) career mentorship and support.

### Residents’ Challenges

#### 1. Meeting Basic Needs

The economic collapse significantly impacted the residents’ ability to meet basic needs, including housing and transportation. Hyperinflation and the Lebanese pound devaluation made it increasingly difficult to meet daily living expenses, with one participant stating: “*the devaluation of the Lebanese pound has led to significant reductions in purchasing power, making it challenging to afford basic necessities such as transportation, food, and housing… (P8).”* Monthly rental rates reached *“400–600$, which is almost half the stipend..*. (P43),” leaving residents with limited ability to manage accommodation and other essential expenses. Logistical barriers emerged as additional demands. For example, commuting to work also created challenges: *“[The] instability of the country, benzene [fuel] shortages in 2021, and closing some main roads […] all made it risky and difficult to reach the hospital (P85).”*

#### 2. Ensuring Wellbeing

The ongoing crises negatively affected the residents’ sense of safety and emotional well-being, leading to emotional strain, fear for personal security, and burnout. Respondents cited political instability and personal financial insecurity as sources of stress and anxiety. One resident shared how the *“Beirut Port blast and its mental toll on everyone (P22)”* intensified the emotional burden, while another reflected on *“severe anxiety due to the political and financial situation (P81).”* Burnout was a significant concern, with residents expressing difficulties maintaining a work-life balance, with one reporting “*The most important challenge was burnout. It was very difficult to deal with that, especially with the expectation that you should show up and perform at your best because ‘everyone is burnt out too’ (P122).”*

#### 3. Maintaining Family and Social Life

Financial and logistical burdens disrupted the residents’ social lives and sense of community, leading to social isolation, reduced familial interaction, and postponement of personal achievements. Respondents reported difficulties establishing stable living situations and achieving personal milestones like marriage and family planning due to financial pressures and uncertainty. One participant highlighted:

*“The financial crisis was a major one. We didn’t have the ability to establish an adequate living (get married, open a house) while in residency and fellowship (P10).”*

#### 4. Fostering Professional Growth and Clinical Experience

The financial collapse and departure of subspecialized faculty challenged the residents’ professional development and academic advancement. The financial crisis restricted academic research and career development opportunities through limited access to *“research funding (P33)”* and *“resources (P55),”* which affected their sense of achievement and self-esteem. For many residents, financial hardships were exacerbated, and it became difficult to afford licensing exams or international travel for fellowships, with one sharing: *“[I] had to go into debt to pay for my Step 2 and 3 exams and to pay for my trip to the USA in preparation for fellowship (P17).”*

Limited access to subspecialized faculty also restricted their clinical exposure to complex cases and procedures: *“[We had] no exposure to modern procedures and complex medical cases after experts left the institution (e.g., advanced heart failure, patients with [left ventricular assist devices] LVADs, solid organ transplant patients) (P77).”* This also negatively impacted their patient care experiences, with one resident admitting that *“not all required interventions could be done on patients (P103).”* However, residents appreciated that: *“[Their institution] made sure to provide the appropriate medical equipment and medication despite the shortage the whole country was facing (P22).”*

#### 5. Fulfilling Career Aspirations

The crises disrupted long-term career aspirations, leading many to reconsider practicing in Lebanon. Uncertainty about the future and limited local opportunities drove residents to seek training and employment abroad. One respondent reflected: *“At the beginning, we were planning to stay in Lebanon and work here, but after the crisis, this became unrealistic (P10),”* and another admitted that *“the situation has made [us] more convinced to leave the country to continue [our] education (P30).”*

### Institutional Support Strategies

#### 1. Essential Financial Support

The institution implemented measures to mitigate these challenges, including salary adjustments in the form of stipend increases and meal tickets. While some residents felt these measures were insufficient saying that *“the stipends should have improved more with the absurd increase in prices everywhere (P43),”* others acknowledged their positive impact. One respondent reported, *“fixing the residents’ and fellows’ salaries helped [us] survive the crisis (P112).”* Residents also suggested *“it would be strategically important to try to provide discounted dorms for the residents that are near the campus (P67).”*

#### 2. Structured Emotional and Psychological Support Programs

The institution offered emotional and psychological support programs. Residents emphasized that *“[Their institution] has made available the resources for emotional and psychological support (free therapy sessions, support groups, etc.) (P43)”*. Another emphasized the need for continuity in support efforts, stating *“structured and regular wellness sessions are needed (P13).”* Despite these resources, many residents expressed the need for better workload management to reduce burnout. As one noted, *“Evening out the load of work by just a bit more would make a big difference (P118),”* and another confirmed *“[I] faced emotional burnout from the overwhelming demands (P122).”* Another resident identified a need for training in *“how to deal with hard families (P20),”* revealing that residents experienced emotional strain from both internal challenges and external stressors, such as complex family dynamics.

#### 3. Faculty and Program Leadership Social Support

Faculty and program leadership support were crucial in fostering a positive work environment.

The residents valued open communication and support from program directors and faculty. One respondent shared *“I was always offered support and guidance from my program director (P51).”* Others valued the availability of leadership to listen and intervene when needed: *“By being good listeners to [our] concerns and finding fast troubleshooting solutions to the little obstacles we would face on a daily basis (P107)*. Another added, *“Support was mainly through encouraging verbal communication (P6*),” underscoring how *“[faculty and chiefs] advice and emotional support helped a lot through the year (P110).”* A resident also noted how *“Chiefs were so supportive in additional to the program director and mentor in guidance throughout the 3 years (P68).”* Others highlighted the importance of role modeling as they observed *“how [faculty] lead and educate juniors and how they deal with us (P55).”*

#### 4. Sustained Career Support and Supervision Amid Crisis

The institution implemented several career support mechanisms to promote professional growth, including research mentorship, scholarship opportunities to attend internal development programs such as *“SHARP [Scholars in Health Research Program] (P33),”* and monetary support offered through the *“[Resident Staff Organization] RSO cabinet (P18)”* to help residents prepare for international examinations.

The remaining faculty maintained bedside teaching and exposure to challenging cases. One resident described how *“[Faculty rounded] with us to see patients under direct supervision and helped us build our clinical skills through patient turnover (P126).”* These interventions had some success: *“I had the best educational experience despite everything happening around. Our Chair, Director, and everyone responsible made sure we received the best educational experience (P1).”*

Despite resource limitations, residents reported that the crisis context provided them with opportunities to learn from their faculty how to manage constraints, adapt to different healthcare settings, treat diverse populations, and provide cost-effective care. They did so by *“learning how to limit the work-up or find alternative medications if any (P107)”* and frequently adjusting treatment plans to patients’ financial constraints, such as medication shortages: *“the financial crisis made my training unique in that I always had to consider the patient’s financial status and learn about all the generic drug products available (P120).”* Another resident explained, *“[Patients exhibited] financial frustration, and [we had] to deal with insurances [tailoring our medical plan accordingly] [in addition to] medication shortages (P22).”*

#### 5. Career Mentorship and Support

The institution facilitated international fellowship transitions through *“providing contacts for* [international] *electives (P44),” “good motivational mentorship and facilitating future training opportunities after residency (P13),”* and providing administrative support for pursuing opportunities abroad through the “*Office of International Programs (P69)”*. The institution also helped residents navigate logistical barriers that could jeopardize timely career progression by coordinating with foreign embassies, closed to the public at that time, to secure “*emergency visa appointments (P25),”* ensuring that residency graduates could begin their fellowships abroad without delay.

## Discussion

This survey of graduating residents in Lebanon revealed that despite compounded crises, they reported high preparedness across all ACGME-I competencies. This paradox of preparedness amid adversity was supported by institutional interventions like salary adjustments, wellness resources, and faculty and leadership support. Many residents credited working in resource-limited conditions with strengthening their Systems-Based Practice skills. However, they faced significant personal and professional challenges, notably economic hardship, which impacted basic needs, social connections, and career planning. Also, maintaining family and social life under such conditions emerged as a particular challenge. While institutional strategies helped mitigate some burdens, others—such as faculty retention—remained largely beyond the institution’s control.

The study extends the literature by examining how overlapping crises shape GME, demonstrating that despite ongoing and extreme duress, residents continuously adapted. The crisis context in Lebanon provided unique opportunities to refine skills in resource management, adaptability, and efficiency. The residents also reported sustained preparedness across all competency domains, highlighting the potential role of institutional support and local adaptation. While financial support measures such as salary adjustments and meal provisions were beneficial, they were insufficient in the face of extreme inflation, highlighting the need for broader structural solutions, such as subsidized housing and more sustainable forms of institutional support.

Similarly, the study underscores the importance of comprehensive well-being support, including proactive workload management [20]. Residents emphasized the need for structural changes beyond duty hour limitations to prevent burnout, reflecting findings from studies on fatigue and work-hour policies [21]. A systematic review showed that the top self-reported drivers of fatigue included excessive duty hours, heavy workloads, and sleep disturbances [22]. However, duty hour restrictions alone, if not implemented alongside broader structural changes, including adjustments to supervision, work load, and educational expectations, have not demonstrated significant benefit to resident burnout or patient safety and may have unintended consequences to educational outcomes [22].

Emotional resilience was also tested by challenging interactions with patients and families, adding to resident stress. This aligns with studies from non-crisis settings that identified patient-related challenges, including conflict with patient families, patient death, and fear of making patient errors, as key stressors for healthcare workers [23]. The crisis likely amplified these stressors, emphasizing the need for targeted training in navigating emotionally charged encounters.

Social support within residency programs emerged as an important buffer against adversity. Faculty mentorship, open communication, and a sense of community enhanced the residents’ social and emotional well-being and fostered belonging within the workplace, consistent with Communities of Practice theory [24]. This theory emphasizes the importance of strong social connections within learning environments [24], and prior research linking social support to increased belonging and mental health benefits [25,26]. Additionally, optimizing limited clinical exposure through high-quality teaching and enhanced faculty mentorship helped sustain educational outcomes despite the loss of subspecialized faculty, aligning with crisis-driven strategies for resource optimization [27]. However, institutional career mentorship support, while beneficial, coincided with broader trends of physician emigration. As previous studies in Lebanon have shown, economic decline, career instability, and political uncertainty remain primary drivers of physician migration, highlighting challenges beyond the institution’s control [28,29].

A key insight from this study is the dominance of economic hardship in resident narratives, surpassing other disruptions like the Beirut Port explosion and the COVID-19 pandemic. This may reflect a pattern of sequential adaptation observed among residents, where they responded to each crisis, recalibrated, and moved forward to face the next. This behavior is reminiscent of Nassim Nicholas Taleb’s concept of antifragility, the idea that some systems grow stronger through continued exposure to stressors and uncertainty [30]. In this context, antifragility can serve as a lens to interpret how prolonged and compounding stressors, rather than isolated events, can shape adaptive responses over time.

It is notable that maintaining social and family life emerged as a significant challenge for residents. This issue reflects not only emotional strain but also a broader need for community-based support. The data suggest that resident well-being during crises depends not only on institutional adaptation but also on external societal support structures. Prior research has shown that work-family support acts as a protective factor against burnout among primary healthcare workers, with career identity mediating this relationship [31]. This suggests that support from others fosters a sense of affirmation and professional value, which in turn might help buffer against external strains. Similarly, studies during the COVID-19 pandemic found that family support significantly influenced burnout levels among healthcare workers [32]. These findings suggest that in times of crisis, resident support must be viewed as a shared responsibility, one that extends beyond the health system to include broader societal engagement.

Our study has important curricular and educational policy implications. The findings demonstrate how crises can provide opportunities for competency development, such as a heightened awareness of resource constraints and improved efficiency in delivering care. Integrating crisis-relevant competencies like resource management and adaptability into medical curricula is needed worldwide. Simulation-based Crisis Resource Management (CRM) training aimed at developing cognitive and interpersonal behavioral skills, including leadership, communication, situational awareness, and resource utilization in chaotic work environments [33] and problem-based learning exercises requiring residents to devise treatment plans within constrained resources can promote adaptability and innovation in patient care, two dimensions recommended by the World Health Organization [34]. Additionally, implementing comprehensive wellness programs, including mental health support, workload management strategies, and peer support networks, should be prioritized [1]. Retention strategies, such as structured career pathways and leveraging the expertise of emigrant professionals through research collaborations, mentorship programs, and visiting faculty roles, are also needed. Finally, institutional policies are needed to support the return of health professionals in order to rebuild the healthcare workforce and facilitate the contribution of emigrant professionals’ expertise to the growth of the local healthcare system.

A key strength of this study is its inclusion of residents from a wide range of specialties, including both medical and surgical fields, enhancing the relevance of findings across diverse training environments. Yet, our results should be viewed in light of some limitations. First, the findings may not be generalizable beyond Lebanon due to unique socio-political factors. Second, the cross-sectional design limits conclusions about long-term impacts. Third, restricting the sample to graduating residents precludes insights into how training level influences crisis adaptation. Fourth, while we included both medical and surgical specialties, future studies may benefit from stratified analyses to explore whether certain types of training programs were more affected than others. Our data did not explicitly reflect individual coping mechanisms and instead centered on environmental and institutional support. Future studies may benefit from exploring both personal and institutional strategies to provide a more comprehensive view of how residents adapt to crises. Lastly, reliance on self-reported perceptions of competence introduces potential bias. Prior literature has shown that physician self-perceptions do not always align with objective assessments, with evidence suggesting that those with lower performance levels may overestimate their abilities [35]. Future research is needed to explore how residents’ perceived competence compares to objective performance data, such as direct observation or standardized assessments.

In conclusion, this study provides valuable insights into the impact of multiple, overlapping crises on GME in Lebanon. While the crises presented significant challenges, the resilience of the GME system and the adaptability of the residents were notable. The findings underscore the importance of prioritizing resident well-being, fostering a supportive learning environment, and developing crisis-relevant competencies to ensure the continued success of medical education in the face of future challenges. Further research is needed to address workload management and assess the incorporation of crisis-driven systems-based skills in healthcare education.

## Data Accessibility Statement

The datasets generated and/or analyzed during the current study are not publicly available but are available from the corresponding author on reasonable request.

## Additional File

The additional file for this article can be found as follows:

10.5334/pme.1721.s1 Supplementary Digital Appendix 1. Questionnaire.

## References

[1] Mahmoud F, Ghadban A, Harhara T, Ibrahim H. Rebuilding graduate medical education after a crisis: Perspectives of medical residents in the United Arab Emirates. Advances in Medical Education and Practice. 2021;507–11. DOI: 10.2147/AMEP.S304659 34040479 PMC8142685

[2] Afshari P, Parast MM, Yazdani S. Do Economic Crises Affect Medical Education? A Narrative Review. Journal of Medical Education for Future Demands. 2024;23(1). DOI: 10.5812/jme-150892

[3] Mladovsky P, Srivastava D, Cylus J, Karanikolos M, Evetovits T, Thomson S, et al. Health policy responses to the financial crisis in Europe: policy summary 5. World Health Organization; 2012.

[4] Al-Shamsi M. Medical education in Iraq: issues and challenges. International journal of medical education. 2017;8:88. DOI: 10.5116/ijme.58b1.c927 28285276 PMC5357542

[5] Bank W. Lebanon: Assessment of the Public Procurement System. World Bank; 2021.

[6] El Khoury FM, Talih F, El Khatib MF, Abi Younes N, Siddik M, Siddik-Sayyid S. Factors associated with mental health outcomes: results from a tertiary referral hospital in Lebanon during the COVID-19 pandemic. Libyan Journal of Medicine. 2021;16(1). DOI: 10.1080/19932820.2021.1901438 PMC8032329 33820499

[7] Islam Z, Gangat SA, Mohanan P, Rahmat ZS, El Chbib D, Marfani WB, et al. Mental health impacts of Lebanon’s economic crisis on healthcare workers amidst COVID-19. The International journal of health planning and management. 2022;37(2):1160. DOI: 10.1002/hpm.3324 34476840 PMC8652701

[8] Msheik El Khoury F, Talih F, Khatib MFE, Abi Younes N, Siddik M, Siddik-Sayyid S. Factors Associated with Mental Health Outcomes: Results from a Tertiary Referral Hospital in Lebanon during the COVID-19 Pandemic. Libyan J Med. 2021;16(1):1901438. DOI: 10.1080/19932820.2021.1901438 33820499 PMC8032329

[9] Hashim HT, Uakkas S, Reda A, Ramadhan MA, Al Mostafa MY. Beirut explosion effects on COVID-19 situation in Lebanon. Disaster Medicine and Public Health Preparedness. 2022;16(5):1703–4. DOI: 10.1017/dmp.2021.56 33588973 PMC8111190

[10] Fares MY, Musharrafieh U, Bizri AR. The impact of the Beirut blast on the COVID-19 situation in Lebanon. Journal of Public Health. 2023;31(4):575–81. DOI: 10.1007/s10389-021-01562-6 34055571 PMC8140319

[11] Zeeni C. Beirut. Anesthesiology. 2020;134(4):661–3. DOI: 10.1097/ALN.0000000000003580

[12] Fakhoury T, Aitken M. Ecologies of Conflict and Coexistence in the Mediterranean: Seeking Refuge in post-war Lebanon. Migrations in the Mediterranean. 2024;173. DOI: 10.1007/978-3-031-42264-5_11

[13] Ghannam AB, Ibrahim HA, Hammoud B, Hamam R. Impact of the economic crisis, COVID-19 and the Beirut explosion on ophthalmology training in Lebanon: an observational cohort survey-based study. BMJ open. 2024;14(3):e075321. DOI: 10.1136/bmjopen-2023-075321 PMC10916134 38448079

[14] Von Elm E, Altman D, Egger M, Pocock S, Gøtzsche P, Vandenbroucke J. The Strengthening the Reporting of Observational Studies in Epidemiology (STROBE) statement: Guidelines for reporting observational studies. Revista Espanola de Salud Publica. 2019;82(3):251–9. 10.1590/s1135-57272008000300002 18711640

[15] Holmboe E, Lobst W. ACGME-I Milestones Guidebook: ACGME-I; 2020 [Available from: https://www.acgme-i.org/globalassets/acgme-international/milestones/assessmentguidebook.pdf

[16] AAMC. AAMC Resident Readiness Survey Program: AAMC; 2023 [Available from: https://www.aamc.org/data-reports/students-residents/report/rrs-project

[17] Bradley EH, Curry LA, Devers KJ. Qualitative data analysis for health services research: developing taxonomy, themes, and theory. Health Serv Res. 2007;42(4):1758–72. DOI: 10.1111/j.1475-6773.2006.00684.x 17286625 PMC1955280

[18] Braun V, Clarke V. Using thematic analysis in psychology. Qualitative Research in Psychology. 2006;3(2):77–101. DOI: 10.1191/1478088706qp063oa

[19] Maslow AH. A theory of human motivation. Psychol Rev. 1943;50(4):370–96. DOI: 10.1037/h0054346

[20] Shanafelt TD, Noseworthy JH, editors. Executive leadership and physician well-being: nine organizational strategies to promote engagement and reduce burnout. Mayo Clinic Proceedings; 2017: Elsevier. DOI: 10.1016/j.mayocp.2016.10.004 27871627

[21] Bauer GF, Hämmig O, Schaufeli WB, Taris TW. A critical review of the job demands-resources model: Implications for improving work and health. Bridging occupational, organizational and public health: A transdisciplinary approach. 2014;43–68. DOI: 10.1007/978-94-007-5640-3_4

[22] Bolster L, Rourke L. The effect of restricting residents’ duty hours on patient safety, resident well-being, and resident education: an updated systematic review. Journal of Graduate Medical Education. 2015;7(3):349–63. DOI: 10.4300/JGME-D-14-00612.1 26457139 PMC4597944

[23] Rink LC, Oyesanya TO, Adair KC, Humphreys JC, Silva SG, Sexton JB. Stressors Among Healthcare Workers: A Summative Content Analysis. Glob Qual Nurs Res. 2023;10:23333936231161127. DOI: 10.1177/23333936231161127 PMC10068501 37020708

[24] Drury J, Carter H, Cocking C, Ntontis E, Tekin Guven S, Amlôt R. Facilitating collective psychosocial resilience in the public in emergencies: Twelve recommendations based on the social identity approach. Frontiers in public health. 2019;7:141. DOI: 10.3389/fpubh.2019.00141 31214561 PMC6558061

[25] Hunderfund ANL, Ardestani BS, Laughlin-Tommaso SK, Jordan BL, Melson VA, Montenegro MM, et al. Sense of Belonging Among Medical Students, Residents, and Fellows: Associations With Burnout, Recruitment Retention, and Learning Environment. Academic Medicine. 2024;10:1097. DOI: 10.1097/ACM.0000000000005892 39348173

[26] Sommerlad A, Marston L, Huntley J, Livingston G, Lewis G, Steptoe A, et al. Social relationships and depression during the COVID-19 lockdown: longitudinal analysis of the COVID-19 Social Study. Psychological medicine. 2022;52(15):3381–90. DOI: 10.1017/S0033291721000039 PMC7844174 33436126

[27] Chick RC, Clifton GT, Peace KM, Propper BW, Hale DF, Alseidi AA, et al. Using Technology to Maintain the Education of Residents During the COVID-19 Pandemic. J Surg Educ. 2020;77(4):729–32. DOI: 10.1016/j.jsurg.2020.03.018 32253133 PMC7270491

[28] Nemr E, Moussallem M, Nemr R, Kosremelli Asmar M. Exodus of Lebanese doctors in times of crisis: a qualitative study. Frontiers in Health Services. 2023;3:1240052. DOI: 10.3389/frhs.2023.1240052 38028945 PMC10643131

[29] Hitti E, Abdul-Nabi SS, Mufarrij A, Kazzi A. Brain drain in Emergency Medicine in Lebanon, building locally and exporting globally. BMC Med Educ. 2025;25(1):138. DOI: 10.1186/s12909-025-06706-w 39875967 PMC11776325

[30] Taleb NN. Lebanon: from Ponzi to Antifragility. Medium 2020 [Available from: https://medium.com/@nntaleb/lebanon-from-ponzi-to-antifragility-c9867eb71998

[31] Yang D, Fang G, Fu D, Hong M, Wang H, Chen Y, et al. Impact of work-family support on job burnout among primary health workers and the mediating role of career identity: A cross-sectional study. Front Public Health. 2023;11:1115792. DOI: 10.3389/fpubh.2023.1115792 36908407 PMC9998699

[32] Setyowati R, Natalia L, Nuraeni R, Zakiyyah K. Relationship between Family Support and the Incidence of Burnout among Healthcare Workers during the COVID-19 Pandemic. Risenologi. 2022;1a(7):31–7. DOI: 10.47028/j.risenologi.2022.71a.329

[33] Lei C, Palm K. Crisis resource management training in medical simulation; 2019. 31869172

[34] WHO. Transforming and Scaling up Health Professional Education and Training; 2013.

[35] Gaeta TJ, Reisdorff E, Barton M, Feldhaus KM, Gausche-Hill M, Goyal D, et al. The Dunning–Kruger effect in resident predicted and actual performance on the American Board of Emergency Medicine in-training examination. J Am Coll Emerg Physicians Open. 2024;5(5):e13305. DOI: 10.1002/emp2.13305 39463809 PMC11502208

