# Supplementary material for: Graduate Medical Education in Lebanon: Challenges, Support, and Adaptation Amid the Compounding Crises
Source: Perspect Med Educ. 2025 Sep 10;14(1):549–59. doi: 10.5334/pme.1721 (PMC12428328; doi:10.5334/pme.1721)
Supplement: Supplementary Digital Appendix 1. — Questionnaire. [file pme-14-1-1721-s1.pdf]

Supplementary Digital Appendix 1. Questionnaire

Demographics

1. **Specialty:**
    a. Anatomic Pathology /Clinical Pathology
    b. Anesthesiology
    c. Dermatology
    d. Diagnostic Radiology
    e. Emergency Medicine
    f. Family Medicine
    g. Internal Medicine
    h. Neurology
    i. Obstetrics & Gynecology
    j. Ophthalmology
    k. Orthodontics
    l. Pediatrics
    m. Psychiatry
    n. Radiation Oncology
    o. Surgery - Neck Surgery
    p. Surgery – Neurosurgery
    q. Surgery - Orthopedic Surgery
    r. Surgery - General Surgery
    s. Surgery - Otorhinolaryngology & Head
    t. Surgery - Plastic & Reconstructive Surgery
    u. Surgery – Urology
    v. Other

2. **Training Program:** Residency, Fellowship

Perceived Preparedness Levels

Please mark your perceived level of preparedness for each of the ACGME-I competencies below.

(Scale: 1 to 3: Unprepared, Prepared, Well Prepared)

3. Patient Care: Ability to provide compassionate, appropriate, and effective care for treating health problems and promoting health.

4. Medical Knowledge: Understanding and application of biomedical, clinical, epidemiological, and social-behavioral sciences in patient care.

5. System-Based Practice: Awareness of and responsiveness to the larger context and system of healthcare, including the ability to effectively call on system resources to provide care that is of optimal value.

6. Interpersonal & Communication Skills: Skills that result in effective information exchange and collaboration with patients, their families, and other health professionals.

7. Professionalism: Residents/Fellows must demonstrate a commitment to carrying out professional responsibilities and an adherence to ethical principles.

8. Practice-Based Learning & Improvement: Ability to investigate and evaluate patient care practices, appraise and assimilate scientific evidence, and continuously improve patient care based on self-evaluation and lifelong learning.

Challenges and Adaptation During Crises

9. What have been your top three challenges as a clinical trainee during the ongoing financial, and political crises in Lebanon? (Focus on specific difficulties or obstacles you have encountered.)

10. In what ways has the leadership at the [redacted] (both at the institutional and program levels) supported you in overcoming these challenges and meeting your needs?

11. Is there anything specific you wish the [redacted] or your program would have done to better support you during these crises? Can you provide specific examples?

12. How have the financial and political crises affected the quality of your training at the [redacted]? Can you provide specific examples of how your educational experience has been impacted?

13. How have the financial and political crises affected your future professional plans?
